# Evaluation of ML-Based Clinical Decision Support Tool to Replace an Existing Tool in an Academic Health System: Lessons Learned

**DOI:** 10.3390/jpm10030104

**Published:** 2020-08-27

**Authors:** Myung Woo, Brooke Alhanti, Sam Lusk, Felicia Dunston, Stephen Blackwelder, Kay S. Lytle, Benjamin A. Goldstein, Armando Bedoya

**Affiliations:** 1 Department of Medicine, Duke University School of Medicine, Durham, NC 27708, USA; myung.woo@duke.edu; 2 Duke Clinical Research Institute, Duke University School of Medicine, Durham, NC 27701, USA; brooke.alhanti@duke.edu (B.A.); Sam.lusk@duke.edu (S.L.); benjamin.a.goldstein@duke.edu (B.A.G.); 3 Duke Health Technology Solutions, Duke University Health System, Durham, NC 27703, USA; felicia.dunston@duke.edu (F.D.); stephen.blackwelder@duke.edu (S.B.); 4 Health Sector Management Program, Duke Fuqua School of Business, Durham, NC 27708, USA; 5 Health System Nursing and Duke Health Technology Solutions, Duke University Health System, Durham, NC 27710, USA; kay.lytle@duke.edu; 6 Department of Biostatistics and Bioinformatics, Duke University School of Medicine, Durham, NC 27708, USA

**Keywords:** machine learning, artificial intelligence, prevention, intervention, pressure injury, falls, clinical decision support

## Abstract

There is increasing application of machine learning tools to problems in healthcare, with an ultimate goal to improve patient safety and health outcomes. When applied appropriately, machine learning tools can augment clinical care provided to patients. However, even if a model has impressive performance characteristics, prospectively evaluating and effectively implementing models into clinical care remains difficult. The primary objective of this paper is to recount our experiences and challenges in comparing a novel machine learning-based clinical decision support tool to legacy, non-machine learning tools addressing potential safety events in the hospitals and to summarize the obstacles which prevented evaluation of clinical efficacy of tools prior to widespread institutional use. We collected and compared safety events data, specifically patient falls and pressure injuries, between the standard of care approach and machine learning (ML)-based clinical decision support (CDS). Our assessment was limited to performance of the model rather than the workflow due to challenges in directly comparing both approaches. We did note a modest improvement in falls with ML-based CDS; however, it was not possible to determine that overall improvement was due to model characteristics.

## 1. Introduction

Tools to support doctors with diagnosis in the effective treatment of patients are as old as empirically-based diagnosis itself, which in the United States dates back to the patient records kept at the bedside in 19th century hospital wards [1]. These clinical decision support (CDS) tools, as they are referred to today, are intended to ensure the right information gets to the right person in an appropriate format for intervention, via the right channel and at the right time in the diagnostic or treatment workflow [2]. While computerized CDS applications were under development as early as the 1960s [3], such systems have become ubiquitous as electronic health record systems have digitized both clinical data and clinical workflow management [4]. CDS tools which predate computerization of clinical workflows were comprised of manually-operated mechanisms such as paper forms, formulas to be worked out by hand or handheld calculator, decision tree-based rule methods and point-scoring algorithms. Initial efforts to integrate CDS into electronic health record (EHR) systems relied heavily on the automation capabilities of the computerized medical record, automating the calculation of these same time-honored scores and calculations [5]. Moving these well-understood tools into the EHR to automate workflow provided some additional efficiency while retaining the familiar and proven tools themselves, lowering resistance to clinician adoption. Recent CDS advancements have often been the result of driving clinical recommendations using an artificial intelligence (AI) mathematical model in place of the grids, scores and rules-based engines commonly underlying previous CDS systems. For the purposes of this discussion, ML will refer to a broad range of machine learning approaches, including deep learning, recombinant and convolutional neural networks, generalized adversarial networks, as well as combinations of these approaches with each other and with other statistical methods. 

CDS tools have shown to improve provider and clinician performance [6,7,8,9]. The tools range in purpose from guiding medical diagnosis to informing therapeutic decisions. As new evidence and guidelines are published, it becomes necessary to update the CDS tools in a timely manner to avoid providing substandard care. However, it can be a significant challenge to modify existing CDS tools that are built upon rules or algorithms that drive their contents [10]. Additionally, with the breadth of data that are generated within the EHR, it has become even more important to keep up to date by incorporating new data as they are generated, which may not be feasible with traditional CDS tools.

With the proliferation of CDS tools, the question has begun to evolve from whether an institution should adopt a particular CDS, to whether a new CDS should replace an existing one. Currently, many of the “newer” CDS tools are machine learning (ML)-based. While such ML-based scores often have superior performance to traditional scores [11,12,13,14,15,16], implementing ML analytic tools poses inherent challenges including incorporation of the tool into the EHR, trainings on tool use, clinical staff interaction with the tool and maintenance. Not surprisingly, there is considerable variability in the implementation and adaption of machine learning models within healthcare systems, as well as in the evaluation of these tools [17,18].

While the recommendations of the CDS tools may appear in the same place within the EHR and may rely on the same range of index values, this change in the behind-the-scenes calculation engine, analyzing patient data and driving recommendations is a significant issue. Where the pre-ML models have demonstrated effectiveness, and the ML models claim high degrees of predictive accuracy, it is far from clear to clinical users: (1) whether the ML-driven models are more accurate than the older approaches or (2) how effective the ML models are compared to established pre-ML practices. These issues confront healthcare systems deploying ML-driven CDS and which have a responsibility to ensure that the CDS delivered into clinical workflows is superior to standard of care in a real world setting. Demonstrating superiority of a CDS tool relative to the existing workflow is challenging but necessary to ensure responsible use of health system resources. Evaluating CDS tools in a straightforward manner is challenging since these tools are implemented within an existing clinical care workflow. Traditional assessments of prediction models are confounded by the necessary prevention measures taken to reduce patient risk. 

This paper illustrates our approach to evaluating two predictive analytic tools deployed in our hospital and how we compared them to an existing simplified CDS tool. These tools were specifically designed to predict the risk of falls and pressure injuries in hospitalized adult patients, as well as provide recommendations to prevent an event. Fall and pressure injury events incur high direct and indirect costs to the hospital entities and to the individual patients [19,20,21,22]. Both tools consisted of two components: first, a dynamic risk score indicating how likely an individual patient would be to have an event and second, suggestion of up to five interventions provided in a ranked-order list to prevent an event. In this paper, we discuss our evaluation approach, contextualize the results and our recommendation to clinical leadership, and provide lessons learned. 

## 2. Methods

### 2.1. Clinical Environment

We implemented the ML-based CDS tools targeted towards nurses caring for adult inpatients in Duke University Health System’s (DUHS) three hospitals: Duke University Hospital (DUH), Duke Regional Hospital (DRH) and Duke Raleigh Hospital (DRAH). Since 2014, DUHS has used a shared Epic-based EHR system. The health system averages 5996 hospitalizations per month with DUH having the largest proportion of patients (62%). DRH accounts for 25% of hospitalizations while DRAH has the remaining 13%.

### 2.2. Clinical Decision Support Tools

The CDS tools evaluated here are targeted at reducing preventable hospital events: patient falls and pressure injury. Before the implementation of the new CDS tools, DUHS used standard of care risk assessment tools. For falls, risk was assessed with an internally developed tool that categorized patients into “high risk” and “not high risk” groups. This assessment was composed of a series of questions and if patient met any criteria, the patient was categorized as ‘high risk’ (Appendix A). For pressure injuries, risk was assessed using the Braden scale [23], which has shown variability in accuracy depending on user and in clinical settings [23,24,25]. We did not stratify patients into subgroups based on Braden scale. We considered patients to be at risk for pressure injury if total score was 18 points or lower. For each of these tools, patients were classified as high or low risk and interventions were implemented for the high risk patients. Nurses received Epic clinical decision support in the form of a best practice advisory reminding them to start a fall risk prevention or pressure injury (ulcer) care plan for the patient. While the care plans included standard interventions, staff were encouraged to individualize them for each patient. 

The new CDS tools were provided by an external vendor and are driven by machine learning. These tools were designed to progressively learn by analyzing large amounts of clinical and non-clinical data including medical diagnoses, medications, nutritional status and socioeconomic information to determine whether a patient is considered “high risk” or “low risk” for an event. Once a patient was categorized into the “high risk” or “low risk” group, the CDS tools recommended up to five rank-ordered interventions that could be implemented by the staff. The tools re-evaluated the patient throughout the hospitalization, updating the current risk status with recommended interventions reflecting the current risk level. 

The staff documented whether a recommendation was ‘implemented’, ‘continued’, ‘completed’, ‘not appropriate for patient’ or ‘unable to implement’. For activities that would continue from shift to shift such as turning the patient or other early mobilization activities, ‘implemented’ was used with the initial documentation and then subsequent documentation used ‘continued’. ‘Completed’ was used for one-time activities such as consulting physical therapy. If a recommendation was not implemented, the staff would select either ‘not appropriate for patient’ to provide feedback to the machine or ‘unable to implement’. 

### 2.3. Workflow Implementation 

After the introduction of the new CDS tools, patients were classified as high or low risk by the CDS tool and individualized; ranked recommendations were provided for the high risk patients. When patients were identified as having low risk for both scoring systems, no interventions were undertaken. For patients deemed high risk by both tools, standard of care interventions were implemented along with any appropriate recommendations proposed by the new CDS. When there was a conflict between the scoring systems, interventions reflecting the highest level of risk were implemented (Table 1). Regardless of the risk score, healthcare providers used clinical judgment to implement interventions and safety measures when deemed necessary.

Prior to full implementation of the CDS tools, nursing leadership and CDS staff developed an adoption plan and online training curriculum. The online training curriculum included a 15 min online module deployed using the learning management system. Tip sheets were available via the user’s Epic learning home dashboard, and emailed to managers and the user group for distribution to staff. Materials were covered in several user group meetings. Immediately after go-live, focus groups and open forums were also used to facilitate education and communication. Targeted training was also performed at specific units post-go-live.

### 2.4. Data Extracted

**Figure 1:**
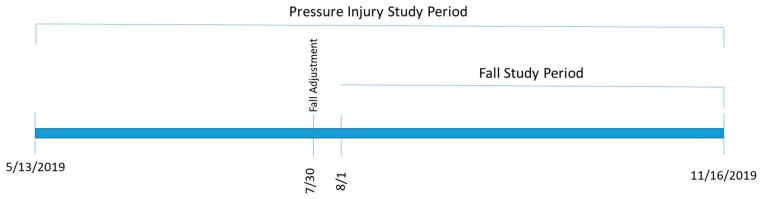
Study timeline.

Data were abstracted from two sources: the DUHS EHR system and our safety reporting system (RL6 by RLDatix) [26]. Hospital events, such as falls and pressure injuries, were manually recorded by the reporting users in RL6. The two sources were merged using medical record number, if available, followed by demographic information. For this study, we defined two study periods. The first period consisted of historic data, which were used to capture background rates of falls and pressure injuries. This covered 1 January 2016 to 12 May 2019. The second period was the implementation period where the new CDS was implemented into our EHR and used by clinical care teams. This covered 13 May–16 November 2019. During this time period, data on events, standard of care risk assignments, CDS risk assignments and CDS recommendations for intervention were collected, resulting in 548 fall events and 187 pressure injury events. After a preliminary assessment, we adjusted the prediction tolerance level for their falls prediction tool on 30 July 2019. Therefore, the evaluation metrics for the falls tool was calculated on data collected from 1 August–16 November 2019, while the pressure injuries evaluation used data from the entire active study period (Figure 1).

### 2.5. Evaluation Design

An ideal evaluation design would have allowed us to evaluate the risk prediction tool and the suggested ranked interventions tool independently. However, this type of evaluation was not possible as the creation of a true control group would necessitate the cessation of current standards of clinical care. Due to these ethical considerations, evaluating the two parts of the CDS tool separately was impossible. This limitation also made direct assessment of the prediction tool with traditional performance metrics (e.g., c-statistics, calibration) impossible. Rather, our evaluation attempted to determine if patient outcomes improved under the CDS tool when compared to the current standard of care, which encompasses both accurate prediction of at-risk patients and effectiveness of the interventions.

Predicted event rates were calculated for both the standard of care tools and the CDS tools and compared to observed event rates. We calculated Kappa statistics to assess agreement between how the standard of care tools and the new CDS tools classified patients into the risk groups. We calculated sensitivity, positive predictive value (PPV), false predicted rate (FPV) and false missed rate (FMR) for both the standard of care and CDS tool predictions at the hospital level. These metrics provide insight into how much the tools were over- or underestimating risk. We also calculated event rates per 1000 patient days and plotted them over the study period to ascertain if the implementation of the CDS tools was resulting in fewer events.

The health system’s determination of whether to continue using the CDS risk assessment tools was based on improvement in patient capture compared to the standard of care approach. The study protocol was approved by the institutional review board of the Duke University Health System.

## 3. Results

### 3.1. Event Rates

During the active evaluation periods, the DUHS hospitals had 112 pressure injuries at a rate of 0.4 pressure injures per 1000 patient days and 209 falls at a rate of two per 1000 patient days. DUH had a higher rate of pressure injuries compared to DRH and DRAH, but fall rates were similar across all three hospitals (Table 2).

### 3.2. Workflow Compliance

We relied on clinical staff to implement the new risk tool as well as the personalized recommendations. We tracked the implementation of the suggested interventions via the clinical EHR interface, where clinical staff could select the suggested CDS interventions that were implemented (and if not implemented, why not). There was significant variation in adoption of the tool between units across the healthcare system. The compliance rate was very low at the start of the study period and increased to around 30% by the end of the active study period. 

### 3.3. Risk Score Comparisons

For the falls tool, we found low concordance between the standard of care and CDS tools with κ = 0.01 in the group with a fall and κ = 0.13 in the group without a fall. Concordance was better for the pressure injury tools with κ = 0.20 in the group with a pressure injury and κ = 0.41 in the group without a pressure injury (Table 3). 

For falls, the standard of care tool assigned about 75% of patients to high risk, while the CDS tool only assigned about 55% of patients to the high-risk category. The standard of care tool tended to overestimate the number of patients at high risk for falls, but still missed 12.4% of falls. By comparison, the CDS tool missed 23.9% of falls but selected 26.4% fewer patients for intervention. For pressure injuries, the Braden score assigned about 28.8% and CDS about 18.6% of patients to high risk. The Braden score missed about 14.3% of pressure injuries. The CDS tool missed 9.8% of pressure injuries and selected 35.6% fewer patients for intervention (Table 3). 

In the context of this evaluation, sensitivity measured the proportion of patients with events who had been assigned high risk for the event. For falls, the standard of care tool had better sensitivity, but for pressure injuries, the CDS tool sensitivity was slightly better (Table 4). Positive predictive value measured the proportion of patients deemed high risk who actually had an event. For both tools, PPVs were similar and low (range: 0.1–2.3%) in falls and pressure injuries. Patients who were predicted to be high risk but did not have an event were either mis-specified or received interventions that prevented the event.

False predicted rate (FPR) is the proportion of patients who were predicted to have an event but did not, out of all patients who did not have events (1-specificity). For falls, the CDS tool performed better than the standard of care tool; however, it is impossible to assess if these patients did not fall because they were actually at low risk or because the interventions were effective at preventing the fall. For pressure injuries, the CDS tool had slightly lower FPR values compared with the Braden scale. The false missed rate (FMR) measured the proportion of patients who were predicted to be low risk but had an event (1-negative predictive value). FMR was low and similar for both pressure injuries and falls (Table 4). 

**Figure 2:**
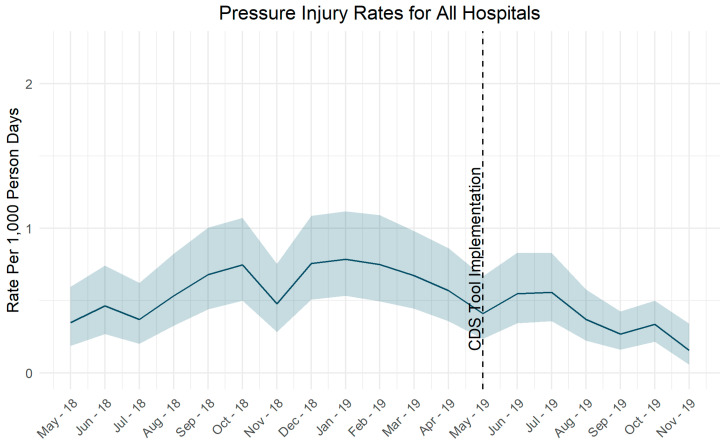
Pressure injury rates over time.

**Figure 3:**
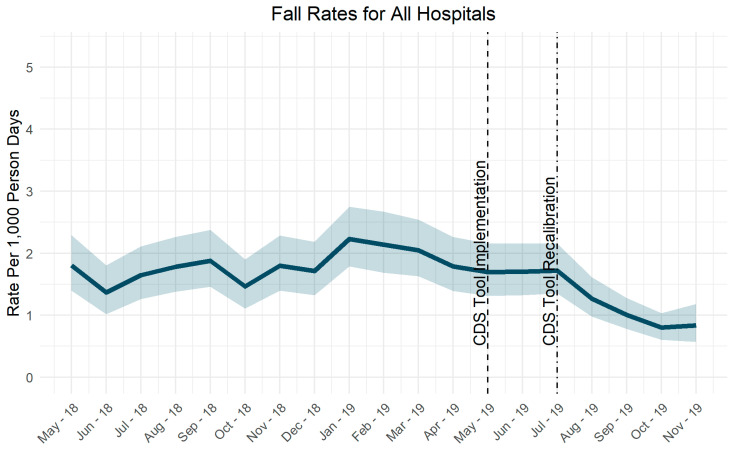
Fall rates over time.

Pressure injury rates did not change significantly after implementation of the CDS tool (Figure 2). Fall rates decreased after adjustment of the risk score tolerance level (Figure 3). 

## 4. Discussion

As CDS tools proliferate, an ongoing question is going to be: are these new tools better? As our experience shows, evaluating a new CDS in the context of an existing CDS is challenging because we cannot “turn off” the current standard of care. This can lead to confusion by providers who interact with the both CDS tools and can make the analytic evaluation more challenging. Currently, many health systems are transitioning from traditional CDS to CDS tools backed by ML. Such tools can enhance clinical care, making their adoption appealing [6]. Therefore, it is imperative to understand the challenges and limitations that might be imposed in order to understand the effects of implementing new CDS tools.

### 4.1. Challenges in Evaluation

#### 4.1.1. Data Matching

The events of interest in this evaluation are stored in the RL6 database rather than Duke Health’s main EHR system, which required merging the two data sources. This process was difficult because RL6 data is entered manually, including the patient’s name, medical record number and demographic data. The manually entered data were more prone to data entry errors, which made merging the event data with other patient-level data complicated. Patient matching is a well-acknowledged issue [26,27]. Others have also noted the challenge of merging patient data from different sources [28]. Often, there is no perfect one-to-one match and some form of fuzzy logic is applied to achieve combining data sources, although with varying success [29,30,31]. We were able to match almost all of the data through medical record number and fuzzy logic matching (seven records out of 2.3 million were unmatchable); however, the data merging process took over a week to complete.

#### 4.1.2. Adjusting Tool Sensitivity

Another challenge in the evaluation was the adjustment of the tolerance level for the CDS falls tool. Post-implementation, it became clear the tool was not sensitive enough and was adjusted. This change effectively removed three months from our evaluation period. 

#### 4.1.3. Adoption

Throughout the implementation and integration of the tool, we had difficulty achieving conformance in utilization of the tools by the end users. Rates of use generally increased over time, but use varied widely across units and hospitals. There were gaps in understanding the intent of the tool and even existence of the tools, which inevitably slowed the adoption for accurate evaluation. The new workflow was complicated as it used multiple risk assessment tools and could at times present different results—one recommending high risk and the other low risk. For the existing standard of care, a care plan was implemented using an organizational standard list of interventions customized to the individual patient. For the CDS tool, the system recommended up to five ranked interventions, with documentation occurring in the flowsheets. This workflow may have been perceived as additional work without justification for some clinical staff, especially when both the existing and new CDS tools indicated the patient was high risk. 

As the active study period progressed, the vendor provided hour long training sessions with a goal of each unit sending at least one designated super user. This additional training focused on understanding the generation of the risk propensity score, and to discuss patient scenarios in which the tool might identify patients at risk when the normal tools did not. In an effort to further improve adoption, the project team reached out to nursing leaders at each of the three hospitals to increase their understanding and discussion of the adoption plans. Rounding on the units offered an opportunity to discuss the functioning and proper use of the new tools, and review clinical scenarios specific to each clinical area. 

Further efforts were made to focus adoption efforts on a subset of units with high rates of falls, pressure injuries or both. Five units were selected for this focused adoption and included two at DUH, two at DRH and one at DRAH. The project team spent time with the unit nurse manager and team leads and any designated super users to increase their understanding of the tool and desired outcomes. All staff were encouraged to complete two new online learning modules, one focused on overall understanding of the tool function for risk propensity and the second focused specifically on pressure injury and non-traditional patient risk. We chose not to limit the evaluation to units with high uptake of the CDS tools as these implementation challenges are part of the real world barriers to success for these types of tools.

#### 4.1.4. Implementation over Standard of Care

To best assess and evaluate the effectiveness of the tools, we would need to control for unintended variables. However, given that the standard of care for these patients was already established, it is infeasible to design an evaluation strategy that would alter the standard workflow and potentially introduce harm to the patients. As such, much thought was put into determining the alternative method of evaluation of the tool without sacrificing the care provided to the patients. Even so, determining the true effect of the CDS tools is difficult to obtain as there were other factors that need to be considered such as education and awareness of the tool to the clinical staff, continuous training and improvements in machine learning algorithm over time. We chose to focus on how the new tool compared to the old tool in terms of ability to predict high-risk patients (measured as patients deemed low risk who had events), number of patients who required intervention and actual event rates over time. While this does not present a perfect evaluation of the new CDS itself, it allows us to understand the potential added value. Moreover, by focusing on clinical performance metrics such as capture and missed rate—as opposed to statistical metrics like area under the curve—we were able to contextualize the differences in actual people detected.

#### 4.1.5. Efficacy

One of the goals of the tool was to improve clinical workflow by providing up to five rank- ordered recommendations to the nursing providers. These recommendations change with patient’s risk score throughout the admission without manually calculating their risks. This had the potential to significantly decrease the number of measures that a nurse would have to implement over the course of the patient’s admission. Unfortunately, we were not able to directly assess the impact on clinical workflow as it was difficult to distinguish the measures implemented under standard of care vs. recommendations provided by the model. 

### 4.2. Decision Based on Evaluation

Due to the challenges of evaluation, our assessment was limited only to performance of the model rather than the entire workflow. Our evaluation noted modest improvement of the falls model over standard of care and no significant difference in the performance characteristics of the pressure injuries model. Although there was modest improvement in the falls model, it was not possible to conclusively state the improvement in characteristics led to the decrease in fall rates. Ultimately, the evaluation team felt that the challenges of evaluation and inability to accurately assess impact outweighed the modest improvement in model characteristics.

## 5. Conclusions

Careful evaluation of clinical risk assessment tools is a key part of their implementation. These evaluations should be approached thoughtfully and take into account the real world constraints surrounding the evaluation of tools in a clinical setting. Some of the constraints we encountered were complicated and messy data structures, challenges in clinical compliance and confounding introduced by using multiple risk assessment tools simultaneously. It is crucial for clinical staff who would be using and carrying out the implementation details to be aligned with the intent of the tools. Without adequate buy-in from clinical staff, it would be incredibly difficult to truly evaluate the impact that a tool may have on the clinical outcome or even any form of evaluation to take place. Regardless of how accurate a tool may be, without support from its end users, usage will be low and resources poured into it lost. 

Running two risk prediction tools simultaneously was confusing to staff and created complications in the evaluation metrics. Being able to designate separate groups to run concurrently would be preferable, however, in this case, impossible since there were already established CDS tools as the standard of care. As more CDS tools become standard of care, the difficulty of comparing newer versions to the standard of care will become commonplace. Evaluation designs will need to focus on how to parse out the impact of the new CDS tool without simply focusing on technical accuracy.

Clinical tools derived from machine learning models will continue to emerge and integrate into healthcare infrastructure. As such, significant weight should be given to understanding the clinical impact such tools would have. This is critical in determining the feasibility of implementation and adaptation by the front-end users and clinical providers, ultimately leading to successful integration of the tool.

## Figures and Tables

**Table 1:** Intervention workflow based on risk scores.

	Low Risk (Standard)	High Risk (Standard)
**Low Risk (CDS)**	No action	Standard of care
**High Risk (CDS)**	CDS recommendations	Standard of care & CDS recommendations

**Table 2:** Events rates per 1000 patient days.

Hospital	Falls	Pressure Injuries
Duke Raleigh	1.8	0.1
Duke Regional	2.1	0.2
Duke University	2.1	0.9
Overall	2.0	0.4

**Table 3:** Concordance of risk scores.

	With Falls (*n* = 209)		Without Falls (*n* = 21,391)	
	Standard Low	Standard High	Standard Low	Standard High
**CDS Tool Low**	6 (2.9%)	44 (21.1%)	3133 (14.6%)	6547 (30.6%)
**CDS Tool High**	20 (9.6%)	139 (66.5%)	2318 (10.8%)	9393 (43.9%)
**Kappa**		0.01		0.13
	**With Pressure Injuries (*n* = 112)**		**Without Pressure Injuries (*n* = 36,983)**	
	**Standard Low**	**Standard High**	**Standard Low**	**Standard High**
**CDS Tool Low**	4 (3.6%)	7 (6.2%)	24,284 (65.7%)	5916 (16%)
**CDS Tool High**	12 (10.7%)	89 (79.5%)	2099 (5.7%)	4684 (12.7%)
**Kappa**		0.20		0.41

**Table 4:** Classification metrics.

Event Type		Standard	CDS Tool
**Falls**	Sensitivity	183/209 (87.6%)	159/209 (76.1%)
	False predicted rate	15,940/21,391 (74.5%)	11,711/21,391 (54.7%)
	Positive predictive value	183/16,123 (1.1%)	159/11,870 (1.3%)
	False missed rate	26/5477 (0.5%)	50/9730 (0.5%)
**Pressure Injuries**	Sensitivity	96/112 (85.7%)	101/112 (90.2%)
	False predicted rate	10,600/36,983 (28.7%)	6783/36,983 (18.3%)
	Positive predictive value	96/10,696 (0.9%)	101/6884 (1.5%)
	False missed rate	16/26,399 (0.1%)	11/30,211 (<0.1%)

## References

[1] Gillum R. (2013). From papyrus to the electronic tablet: A brief history of the clinical medical record with lessons for the digital age. Am. J. Med..

[2] Campbell R.J. (2013). The five rights of clinical decision support: CDS tools helpful for meeting meaningful use. J. AHIMA.

[3] Shortliffe E.H. (2014). Biomedical Informatics: Computer Applications in Health Care and Biomedicine.

[4] Alther M., Reddy C.K., Reddy C.K., Aggarwal C.C. (2015). Clinical decision support systems. Healthcare Data Analytics.

[5] Miller R.A., Waitman L.R., Chen S., Rosenbloom S.T. (2005). The anatomy of decision support during inpatient care provider order entry (CPOE): Empirical observations from a decade of CPOE experience at Vanderbilt. J. Biomed. Inform..

[6] Jaspers M.W., Smeulers M., Vermeulen H., Peute L.W. (2011). Effects of clinical decision-support systems on practioner performance and patient outcomes: A synthesis of high-quality systemic review findings. J. Am. Med. Inform. Assoc..

[7] Garg A.X., Adhikari N.K.J., McDonald H., Rosas-Arellano M.P., Devereaux P.J., Beyene J., Sam J., Haynes R.B. (2005). Effects of computerized clinical decision support systems on practitioner performance and patient outcomes. JAMA.

[8] Jenders R.A. (2017). Advances in clinical decision support: Highlights of practice and the literature 2015–2016. Yearb. Med. Inform..

[9] Sim I., Gorman P., Greenes R.A., Haynes R.B., Kaplan B., Lehmann H.P., Tang P.C. (2001). Clinical decision support systems for the practice of evidence-based medicine. J. Am. Med. Inform. Assoc..

[10] Ash J.S., Sittig D.F., Campbell E.M., Guappone K.P., Dykstra R.H. (2007). Some unintended consequences of clinical decision support systems. AMIA Annu. Symp. Proc..

[11] O’Brien C., Goldstein B., Shen Y., Phelan M., Lambert C., Bedoya A.D., Steorts R.C. (2020). Development, implementation, and evaluation of an in-hospital optimized early warning score for patient deterioration. MDM Policy Pract..

[12] Kia A., Timsina P., Joshi H.N., Klang E., Gupta R.R., Freeman R., Reich D.L., Tomlinson M.S., Dudley J.T., Kohli-Seth R. (2020). MEWS++: Enhancing the prediction of clinical deterioration in admitted patients through a machine learning model. J. Clin. Med..

[13] Churpek M.M., Yuen T.C., Winslow C., Meltzer D.O., Kattan M.W., Edelson D.P. (2016). Multicenter comparison of machine learning methods and conventional regression for predicting clinical deterioration on the wards. Crit. Care Med..

[14] Morgan D.J., Bame B., Zimand P., Dooley P., Thom K.A., Harris A.D., Bentzen S., Ettinger W., Garrett-Ray S.D., Tracy J.K. (2019). Assessment of machine learning vs standard prediction rules for predicting hospital readmissions. JAMA Netw. Open.

[15] Ashfaq A., Sant’Anna A., Lingman M., Nowaczyk S. (2019). Readmission prediction using deep learning on electronic health records. J. Biomed. Inform..

[16] Avati A., Jung K., Harman S., Downing L., Ng A., Shah N. (2017). Improving palliative care with deep learning. IEEE Int. Conf. Bioinform. Biomed..

[17] Kelly C., Karthikesalingam A., Suleyman M., Corrado G., King D. (2019). Key challenges for delivering clinical impact with artificial intelligence. BMC Med..

[18] Magrabi F., Ammenwerth E., McNair J.B., De Keizer N.F., Hyppönen H., Nykänen P., Rigby M., Scott P., Vehko T., Wong Z.S.-Y. (2019). Artificial intelligence in clinical decision support: Challenges for evaluating AI and practical implications. Yearb. Med. Inform..

[19] Stevens J.A., Corso P.S., Finkelstein E.A., Miller T.R. (2006). The costs of fatal and non-fatal falls among older adults. Inj. Prev..

[20] Padula W.V., Delarmente B.A. (2019). The national cost of hospital-acquired pressure injuries in the United States. Int. Wound J..

[21] Padula W.V., Pronovost P.J., Makic M.B.F., Wald H.L., Moran D., Mishra M.K., Meltzer D.O. (2018). Value of hospital resources for effective pressure injury prevention: A cost-effectiveness analysis. BMJ Qual. Saf..

[22] Wong C.A., Recktenwald A.J., Jones M.L., Waterman B.M., Bollini M.L., Dunagan W.C. (2011). The cost of serious fall-related injuries at three Midwestern hospitals. Jt. Comm. J. Qual. Patient Saf..

[23] Bergstrom N., Braden B.J., Laguzza A., Holman V. (1987). The Braden scale for predicting pressure score risk. Nurs. Res..

[24] Lima-Serrano M., González-Méndez M., Martín-Castaño C., Alonso-Araujo I., Lima-Rodríguez J. (2018). Validez predictiva y fiabilidad de la escala de Braden para valoración del riesgo de úlceras por presión en una unidad de cuidados intensivos. Med. Intensiva.

[25] Roca-Biosca A., Rubio-Rico L., Fernández M.D.M., Grau N.G., Garijo G.T., Fernández F.G. (2017). Predictive validity of the Braden scale for assessing risk of developing pressure ulcers and dependence-related lesions. J. Wound Care.

[26] RLDatix. https://rldatix.com/.

[27] Just B.H., Marc D., Munns M., Sandefer R. (2016). Why patient matching is a challenge: Research on master patient index (MPI) data discrepancies in key identifying fields. Perspect. Health Inf. Manag..

[28] Hillestad R., Bigelow J.H., Chaudhry B., Dreyer P., Greenberg M.D., Meili R.C., Ridgely M.S., Rothenberg J., Taylor R. (2008). Identity Crisis: An Examination of the Costs and Benefits of a Unique Patient Identifier for the U.S. Healthcare System.

[29] Heflin E., Thornton S., Smith R. (2014). An Approach to Understanding and Resolving Inter-Organizational Patient Matching.

[30] Dexheimer J.W., Beal S.J., Divekar P., Hall E.S., Patel V., Greiner M.V. (2019). Automated patient linking for electronic health record and child welfare databases. J. Technol. Hum. Serv..

[31] Duggal R., Khatri S.K., Shukla B. Improving patient matching: Single patient view for clinical decision support using big data analytics. Proceedings of the 2015 4th International Conference on Reliability, Infocom Technologies and Optimization (ICRITO).

